# Texas State Society of Social Hygiene

**Published:** 1912-08

**Authors:** 


### Texas State Society of Social Hygiene.

The Texas State Society of Social Hygiene was organized as an integral part of the Texas Medical Association to carry out a distinct work. The particular work thus delegated to this society is the matter of educating the public along the lines of sex hygiene and medical sociology. By co-operation with the laity it was thought this could best be done by a separate organization, which would in now way infringe upon the routine work of the parent association. At the Waco meeting, in May last, the State Association passed a resolution instructing its Committee on Legislation to co-operate with this society in formulating such laws as may be deemed proper to present to the next Legislature bearing upon the question under discussion.

With this end in view, the officers of this society have availed themselves of the opportunity offered by the "Red Back" (TEXAS MEDICAL JOURNAL) to use its pages as a medium of communication with the public, and will endeavor to make this department so interesting and instructive that the JOURNAL will find its way into every home in the State. Indeed, we feel this is an auspicious opportunity and believe it to be a propitious time for the work of the society.

Truth needs no apology, but conventionalities must always be conformed to, and hence the things that need be said can best be said through these columns. The great principles of human interest that are now foremost in thought and which underlie society will be discussed in no mincing tone, but in an open, candid manner that truth requires, to the end that our race may be benefited and the present generation helped to discharge the inherent duty it owes to the next generation; to teach the evolution of the mind, body and character for the better performance of greater usefulness and happiness, ever striving for—

> "One God, one law, one element,
> And one far off divine event,
> To which the whole creation moves."

